# New codon 198 β-tubulin polymorphisms in highly benzimidazole resistant *Haemonchus contortus* from goats in three different states in Sudan

**DOI:** 10.1186/s13071-020-3978-6

**Published:** 2020-03-02

**Authors:** Khalid M. Mohammedsalih, Jürgen Krücken, Amna Khalafalla, Ahmed Bashar, Fathel-Rahman Juma, Adam Abakar, Abdalhakaim A. H. Abdalmalaik, Gerald Coles, Georg von Samson-Himmelstjerna

**Affiliations:** 1 Faculty of Veterinary Science, University of Nyala, Nyala, Sudan; 2 Institute for Parasitology and Tropical Veterinary Medicine, Freie Universität Berlin, Robert-von-Ostertag-Str. 7–13, 14163 Berlin, Germany; 3 Faculty of Veterinary Medicine, University of Khartoum, PO Box 32, Khartoum North, Sudan; 4 Faculty of Medical Laboratory Sciences, University of Gezira, PO Box 20, Wad Medani, Sudan; 5 Ubley Biologics, PO Box 170, Ubley, Bristol, BS40 6JA UK

**Keywords:** Benzimidazole resistance, Molecular mechanisms, Trichostrongyle, Small ruminants, Sudan

## Abstract

**Background:**

Benzimidazole (BZ) resistance in gastrointestinal nematodes is a worldwide problem for livestock production, particularly in small ruminants. Assignment of the emergence of resistance using sensitive and reliable methods is required to adopt the correct strategies for control. In Sudan, BZ resistant *Haemonchus contortus* populations were recently reported in goats in South Darfur. This study aimed to provide additional data regarding albendazole efficacy and to describe the prevailing molecular BZ resistance mechanisms.

**Methods:**

Faecal egg count reduction and egg hatch tests (EHT) were used to evaluate albendazole efficacy in three different areas of South Darfur using naturally (Rehed Al-Birdi and Tulus) and experimentally infected (Tulus and Um Dafuq) goats. Using samples from Central, East and South Darfur, pyro- and Sanger sequencing were used to detect the polymorphisms F167Y, E198A and F200Y in *H. contortus* isotype 1 β-tubulin in DNA extracted from pooled third-stage larval (L3) samples (*n* = 36) on days 0 and 10 during trials, and from pooled adult male *H. contortus* (treated goats, *n* = 14; abattoirs, *n* = 83) including samples from populations previously found to be resistant in South Darfur.

**Results:**

Albendazole efficacies at 5, 7.5 and 10 mg/kg doses were 73.5–90.2% on day 14 in natural and experimental infections while 12.5 mg/kg showed > 96.6% efficacy. EC_50_ in the EHT were 0.8 and 0.11 µg/ml thiabendazole in natural and experimental infection trials, respectively. PCRs detected *Haemonchus*, *Trichostrongylus* and *Cooperia* in L3 samples from albendazole-treated goats. *Haemonchus contortus* allele frequencies in codons 167 and 200 using pyrosequencing assays were ≤ 7.4% while codon 198 assays failed. Sanger sequencing revealed five novel polymorphisms at codon 198. Noteworthy, an E198L substitution was present in 82% of the samples (L3 and adults) including all post-treatment samples. Moreover, E198V, E198K and potentially E198I, and E198Stop were identified in a few samples.

**Conclusions:**

To our knowledge, this is the first report of E198L in BZ resistant *H. contortus* and the second where this is the predominant genotype associated with resistance in any strongyle species. Since this variant cannot be quantified using pyrosequencing, the results highlight important limitations in the general applicability of pyrosequencing to quantify BZ resistance genotypes.
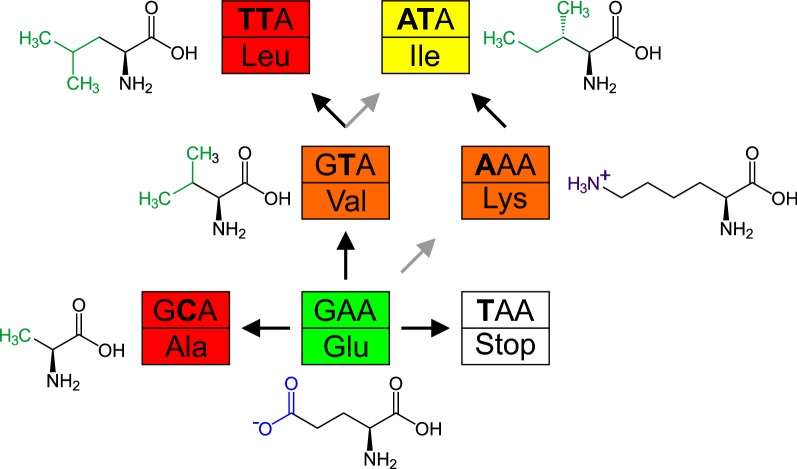

## Background

Parasitic nematodes have a major impact of the health on both humans and animals, particularly in developing countries [1]. The economic impact due to infection with these parasites, mainly gastrointestinal nematodes (GINs), is high, particularly in livestock [2], e.g. estimated to be around 318 million dollars annually in Australia [3]. This impact led to the routine use of anthelmintics in veterinary medicine over several decades [4]. As a consequence of frequent and occasionally indiscriminate use of anthelmintics, parasite populations have developed anthelmintic resistance (AR). This has become widespread in multiple parasites of animals, and threatens the efforts of parasite control in both human and veterinary medicine [5, 6].

Anthelmintic resistance is already a significant problem in veterinary medicine [7], and currently is suspected to be developing in nematodes that infect humans [8]. The economic impact of AR in farm animals is caused by direct production losses such as losses due to clinical and subclinical disease despite treatment, costs for testing of anthelmintic efficacy and the use of new, more expensive classes of anthelmintics [9]. Many studies have been undertaken to determine the factors which increase the development of AR and how they might be managed [10]. To address the widespread nature of AR, detailed knowledge of the molecular mechanisms of resistance is required to improve diagnostic tools for field surveys and to revise the anthelmintic treatment strategies. The most widely used method for detection of AR of animal parasites is the faecal egg count reduction test (FECRT) that can be employed for testing the efficacy for all anthelmintic drug classes, e.g. benzimidazoles (BZs) [11]. Additionally, various *in vitro* tests have been described, such as the egg hatch test (EHT) and the larval development test [11, 12]. Some of the limitations of the current *in vivo* and *in vitro* diagnostic tests for AR could be potentially overcome through the use of molecular techniques that detect specific mutations associated with the resistance phenotype. In particular, such tests are potentially more sensitive, allowing the earlier detection of resistance [7].

For BZ anthelmintics, the most widely used anthelmintics for helminth control in animals, the mode of action as well as the resistant mechanisms are comparatively well understood. At the biochemical level, BZs have been shown to inhibit the polymerization of microtubules [13], and mutagenesis screens in *Saccharomyces cerevisiae* [14] and *Caenorhabditis elegans* [15] identified numerous β-tubulin mutant alleles that are able to confer resistance to BZs. The first identified single nucleotide polymorphism (SNP) in a parasitic nematode associated with BZ resistance was the F200Y polymorphism (T**T**C → T**A**C; resulting in phenylalanine to tyrosine substitution in codon 200) in the isotype-1 β-tubulin gene of *Haemonchus contortus*, a parasitic nematode of small ruminants [16]. Two additional variants associated with BZ resistance have been described at codon positions of isotype-1 β-tubulin gene; F167Y (T**T**C → T**A**C) [17] and E198A (G**A**A → G**C**A) [18]. F200Y, when compared to F167Y and E198A, has been thought to play the most important role in BZ resistance in various parasitic nematodes derived from different regions of the world [7, 19]. Recently, a new substitution was described at codon 198 (E198L: **GA**A → **TT**A) in *Teladorsagia circumcincta*, this mutation was first described by Redman et al. [19] and subsequently shown to be widespread in this parasite by Avramenko et al. [20]. In fungi, all four variants have been demonstrated to confer BZ resistance [21–23].

Molecular techniques have been established for qualitative detection and quantification of allele frequencies for the three substitutions F167Y, E198A and F200Y using conventional PCR, real time PCR, pyrosequencing assays, droplet digital PCR and deep amplicon sequencing in various parasitic nematodes including *H. contortus*, *Trichostrongylus colubriformis*, *Cooperia oncophora*, *Ostertagia ostertagi* and *T. circumcincta* [20, 24–28]. Pyrosequencing assays are now widely used to detect resistance predicting alleles in DNA extracted from field samples using pooled adult worms or larval stages [26, 29, 30].

In Africa, only few molecular studies have been conducted to understand the reduction in efficacy of BZ anthelmintics in parasitic nematodes of both humans and animals. Studies analysing *Haemonchus* spp. populations from some regions in Africa mainly identified the presence of two variants; E198A and F200Y in the isotype 1 β-tubulin gene. Ghisi et al. [18] found E198A to correlate with resistance to BZs in *H. contortus* field isolates from South Africa. Arafa et al. [31] revealed F200Y as a frequent genetic marker for resistance to BZs in *H. contortus* samples from Egypt. Recently in Mozambique, F200Y was detected frequently in BZ-resistance in *H. contortus* in smallholder goat farms [32]. Another study on adult *H. placei* samples from slaughterhouses in Nigeria did not detect any resistance-associated genotypes at codons 167, 198 and 200 [33].

Phenotypically BZ resistant *H. contortus* populations (FECRT: 75–87%; EHT: 0.12–0.24 µg/ml thiabendazole) were very recently reported for the first time in goats in Sudan from the State South Darfur [34]. The present study aimed to identify additional BZ resistant populations and to understand the mechanisms of BZ resistance, through detecting the changes in isotype 1 β tubulin sequences, in three different Darfur States of Sudan.

## Methods

### Study location and design

The study was conducted in three different Darfur states in southwestern Sudan. In South Darfur (11.30°N, 24.40°E), the following six areas were selected: Buram, Kass, Nyala, Rehed Al-Birdi, Tulus and Um Dafuq. In the remaining two states, East (11.10°N, 26.30°E) and Central (12.23°N, 23.18°E) Darfur, only the capital cities, Zalingei (Central Darfur) and Ed Daein (East Darfur) were represented (Additional file 1: Figure S1). The three states are in a savannah zone with the presence of a very long dry season with no rain at all and only a single rainy season, June-November (range 377–546 mm), with mean minimum and maximum temperatures of 20–36 °C and mean minimum and maximum relative humidity of 28.3–56.7% [35]. The open grazing system is the main husbandry system, where the pastures are often dominated by abo-asabei grass (*Dactyloctenium aegyptium*). In these states, the desert goat is the predominant breed, but cross-breeds are also widespread [36].

In South Darfur, BZ resistance in goats (*Capra hircus*) was evaluated using both phenotypic, FECRT and EHT, and molecular techniques, while in East and Central Darfur, the resistance was only studied at the molecular level using samples from abattoirs. Since phenotypic BZ resistance was recently reported in goats from Kass and Nyala [34], samples from these areas collected during the previously reported field trials (June 2015–December 2016) were used here and studied at the molecular level. No previous data were available for the other areas. Therefore, BZ resistance was evaluated in Rehed Al-Birdi, Tulus and Um Dafuq using both phenotypic and molecular approaches, this was not possible in Buram due to limitations in infrastructure, where therefore only molecular data were obtained. The trials and sample collections for molecular analysis were performed between June 2015 and January 2018.

The size of goat farms in the selected areas is always very small (mostly only up to ten animals per owner), and animals belonging to different owners frequently share pastures. Therefore, BZ susceptibility was evaluated phenotypically at region level, while the samples for molecular analysis were pooled based on the farms. The design of the present study and the origin of all samples is summarised in Additional file 2: Table S1.

### Faecal egg count reductions in goats naturally infected with strongyle nematodes

In autumn 2016, trials for evaluating albendazole efficacy were conducted in two different South Darfur study areas, Rehed Al-Birdi and Tulus. A total of 328 goats of both sexes and in different age groups that had not received any anthelmintic treatment for at least one month were selected for collection of faecal samples to be tested for the presence of GIN infections. Twenty-nine farms [Rehed Al-Birdi (*n* = 15) and Tulus (*n* = 14)] with at least five animals per farm [34] were screened. Before sampling, the age (grouped into young (< than 1 year-old) and adults (≥ 1 year-old), based on dentition [37]) and the sex of the goats were determined.

Goats positive for infection with strongyle nematodes were classified into groups of egg shedding intensity: low (< 500 eggs per gram (epg) faeces); moderate (500–2000 epg); or high (> 2000 epg) intensity [38]. Accordingly, 127 moderate to highly infected goats [Rehed Al-Birdi (*n* = 77) and Tulus (*n* = 50)] of both sexes and age groups were selected for a FECRT and grouped into control and treated groups using the same strategy as recently described [34]. Two commercial albendazole brands were used [Endospec 2.5% (batch No: XMF031A; Bimeda, Llangefni, Wales); and Albex^®^ 10% w/v oral suspension (batch No. H30275; Chanelle, Hungerford, UK)]. Before albendazole treatment, the body weight of each animal was determined using a spring balance scale (maximum weight 100 kg) in young goats and a linear body measurement approach in adults which is based principally on the measurement of heart girth and body length (in centimetres) [39]. The approach was found to be accurate in estimation of live weight of Sudanese goats [40]. In these cases, the body weight was calculated using the formula: Heart girth × heart girth × body length/600 = animal weight in kilograms.

Since the 5 mg/kg body weight (bw) (ovine dose) is still prescribed on the package labels of different albendazole commercial brands in Sudan to be used in the treatment of GIN infected goats, and the farmers in South Darfur usually retreat their animals with the same anthelmintic at the same dose again after 14 days [34], here different treatment strategies were adopted and dosages to obtain information regarding (i) the BZ resistance status by administering the appropriate dose of albendazole for goats (10 mg/kg bw) [41]; (ii) the efficacy of the 5 mg/kg bw recommended on the label of the drugs in Sudan; (iii) the differences in efficacy of albendazole at doses of 5, 7.5, 10 and 12.5 mg/kg bw to remove some economically important GINs of goats based on genus-specific PCR; (iv) the variants in allele frequencies at the three codon positions associated with BZ resistance when the 5 mg/kg albendazole dose recommended for goats in Sudan was compared to 7.5, 10 and 12.5 mg/kg bw; and (v) to measure the effects of repeated treatments with 5 mg/kg bw as frequently used by local farmers on frequencies of resistance alleles.

In both study areas, 37 goats [Rehed Al-Birdi (*n* = 25) and Tulus (*n* = 12)] received 10 mg/kg bw albendazole and 35 goats [Rehed Al-Birdi (*n* = 8) and Tulus (*n* = 27)] were treated with 5 mg/kg bw, while the other treatment strategies were either performed in each of the two study areas. In Rehed Al-Birdi, 10 goats received 7.5 mg/kg bw and 13 received 12.5 mg/kg bw. In Tulus, 12 goats from the 5 mg/kg trial with an epg ≥ 400 [30] were retreated with 5 mg/kg bw on day 14. For a better understand of albendazole efficacy [42, 43], here it was evaluated using faecal samples before treatment (day 0) as well as 8 and 14 days after administration of albendazole. All goats involved in the study stayed in their flocks throughout the experiment and remained there after the experiment was finished.

### Faecal egg count reduction in goats experimentally infected with local *H. contortus* isolates

Parallel to our main field trials in goats naturally infected with GINs, the efficacy of the locally recommended dose rate of 5 mg albendazole/kg bw was evaluated also in goats experimentally infected with local *H. contortus* isolates. Two separate experimental infection trials using two different *H. contortus* isolates from Tulus and Um Dafuq were conducted in the facilities of the Faculty of Veterinary Science, University of Nyala, Nyala, Sudan. For each trial, *H. contortus* females were isolated from abomasa (*n* = 50) of goats in the respective abattoirs. The same methods and protocols for preparation of infective larvae from local parasites, infection, observation and treatment of the infected animals were used [34]. For each trial, sixteen GIN-free male goats 3–6 months of age were individually infected with an oral dose of 150 (Tulus) or 200 (Um Dafuq) *in vitro* cultured *H. contortus* L3/kg bw [44] and from day -10 of the trial, they were regularly tested for shedding of nematode eggs. On day 0 (day 23 post-infection) half of the goats (*n* = 8) were treated with 5 mg albendazole/kg bw while the control group was left untreated. Faecal samples were collected on days 0, 8 and 14, respectively. After the experiments were completed, goats used in these trials were slaughtered at Nyala abattoir by the halal method [45] allowing for withdrawal times for drugs used.

### Faecal sample analysis

#### Faecal egg counts

Individual rectal faecal samples were collected in plastic bags, labelled and stored at 4 °C for a maximum of 24 h. Samples from the experimental infection trials (Tulus and Um Dafuq) were analysed at the Laboratory of Parasitology, Faculty of Veterinary Science, University of Nyala, while those from natural infection trials (Rehed Al-Birdi and Tulus) were analysed directly in the field. For counting, the Mini-FLOTAC method and saturated sodium chloride solution as flotation medium were used with a sensitivity of 5 epg [46]. Helminth eggs were identified according to Bowman [47].

#### Faecal cultures

Pooled faecal cultures were prepared on the farm level from naturally infected goats in each study area on days 0 and 10. In labelled plastic jars, the cultures were incubated at 22–27 °C with daily moistening using distilled water for 8 days. The Baermann funnel method was used for harvesting L3 larvae. Then, the percentages of strongyle nematode groups was determined microscopically by differentiating 100 L3 into (i) *Haemonchus* spp.; (ii) *Trichostrongylus* spp.; and (iii) *Oesophagostomum/Chabertia* spp. [47, 48].

#### Egg hatch test

The EHT was performed as described in the guidelines of the World Association for the Advancement of Veterinary Parasitology (WAAVP) [11, 43]. The same approach was used as recently detailed in Mohammedsalih et al. [34].

### Statistical analysis

Albendazole efficacy was evaluated (on a regional basis) using the FECRT by comparing faecal egg count data from day 0 with data from days 8 and 14, respectively. Alternatively, data from days 8 or 14 were compared between control and treatment groups. Since data from the same animal before and after treatment and from different groups (treatment *vs* control) were available, paired and unpaired calculations of FECRs with 95% confidence intervals (CIs) were performed [11, 49]. To consider data structure (paired *vs* unpaired) and high precision of egg counts achieved by Mini-FLOTAC compared to e.g. McMaster, the R package eggCounts version 1.1-1 developed by Wang et al. [50] was used with the zero-inflation option.

The results of the FECRT were interpreted according to Coles et al. [11] and Lyndal-Murphy et al. [51]. Resistance to BZs was assumed when the FECR and its upper 95% CI were < 95% and the lower 95% CI was < 90%. Parasites were considered to be susceptible when the FECR > 95% and its lower 95% CI was > 90%. Otherwise, the FECRT was considered to be inconclusive.

For the EHT, EC_50_ values were calculated using four-parameter logistic regression analysis in GraphPad Prism version 6.01. Resistance to BZs was assumed when the calculated EC_50_ was higher than 0.1 µg/ml thiabendazole [11, 43].

### Parasite material for molecular analyses

#### Third-stage larvae

To identify genotypes conferring BZ resistance in *H. contortus* in different South Darfur study areas, L3 larvae from previous trials (Kass, Nyala (Domaia and Majok subareas) [34]) as well as the trials described in the present study (Rehed Al-Birdi and Tulus) were prepared as pools on the farm level, based on the day of the treatment (i.e. day 0 or day 10) and the tested dose of albendazole (i.e. 5, 7.5, 10 or 12.5 mg/kg bw). In total, 40 samples each containing at least ~ 1000 nematode L3 were collected. Each sample consisted of L3 pooled from at least 4 animals. Of the 40 samples, 15 were from day 0 and 25 from day 10 after treatment, including 11 pairs of samples constituting pools of animals before and after treatment. The strategy used for preparation of pooled samples basically depended on the number of animals on each farm; if the number was less than 4 animals, pooled samples were prepared from animals, mainly post-treated, derived from 2 or more farms that shared the same pasture for grazing. If four or more animals from the same group [i.e. control or treatment (receiving the same dose of albendazole)] were present on a farm, L3 were pooled only from this farm. Paired pools were samples of L3 obtained from 11 farms. Nematode L3s were preserved in 70% ethanol.

#### Adult male *Haemonchus* spp.

At the end of the natural/experimental infection trials in South Darfur (day 14 after treatment with 5 or 10 mg/kg albendazole), 14 goats (Kass: *n* = 3; Nyala: *n* = 3; Rehed Al-Birdi: *n* = 2; Tulus: *n* = 4; and Um Dafuq: *n* = 2) shedding the highest strongyle epg were slaughtered at the abattoir, practicing the halal method [45], then abomasa were isolated and all adult male *Haemonchus* spp. (11–84 from each abomasum) were collected. In addition, 83 goat abomasa from animals not involved in the previous studies and with unknown history of anthelmintic treatment, were chosen by convenience sampling and all adult male *Haemonchus* spp. (10–97 from each abomasum) were isolated and pooled for each individual goat. Of these 83 pools of adult male *Haemonchus* spp., 11 were collected from Central Darfur, 11 from East Darfur and 61 from South Darfur. Samples from South Darfur came from Buram (*n* = 11), Kass (*n* = 9), Nyala (*n* = 20), Rehed Al-Birdi (*n* = 8) and Tulus (*n* = 13). The abattoir in each study area in the three States, was visited once, and all goat abomasa were inspected for the presence of *Haemonchus* spp. before isolation of adults from the positive abomasa. Nyala has two main abattoirs and both were visited on two different days. Worms were preserved in 80% ethanol.

### Genomic DNA isolation

Pooled L3 samples were washed five times with distilled water to remove ethanol. Genomic DNA was extracted using NucleoSpin^®^ Soil DNA extraction kit (Macherey-Nagel, Düren, Germany). Larvae were homogenised using NucleoSpin^®^ Bead Tubes with ceramic beads in the presence of SL1 lysis buffer (Macherey-Nagel) using a speedmill P12 (Analytik Jena, Jena, Germany). The lysates were transferred into new 1.5 ml Eppendorf tubes and DNA was extracted as instructed by the manufacturer. DNA was eluted in 50 µl elution buffer and stored at − 20 °C until used for further analysis.

Pooled adult males were treated as above and DNA was extracted using the NucleoSpin^®^ DNA Tissue extraction kit (Macherey-Nagel). Worms were homogenised using pestles in 1.5 ml microcentrifugation tubes containing 200 µl lysis buffer with proteinase K and incubated at 56 °C overnight with shaking (300× *rpm*). Then, lysates were transferred onto NucleoSpin^®^ columns followed by DNA purification as recommended by the manufacturer. DNA was eluted in 100 µl elution buffer and stored at − 20 °C.

### Genus-specific PCRs

The genus-specific PCRs described by Demeler et al. [52] were used to detect the presence of the economically important GIN of the genera *Haemonchus*, *Trichostrongylus*, *Cooperia*, *Teladorsagia* and *Ostertagia* in L3 samples from goats in South Darfur before and after treatment with albendazole. PCRs were used to amplify partial internal transcribed spacer 2 (ITS2) regions using the specific primers listed in Additional file 3: Table S2. The reaction contained 0.2 mM dNTPs, 0.25 mM of each primer, 0.4 U Phusion Hot Start II High-Fidelity DNA polymerase (Thermo Fisher Scientific, Waltham, USA) and 2 µl (20–40 ng) template DNA in 20 µl 1× HF buffer. Reaction conditions included an initial denaturation at 98 °C for 30 s, followed by 40 cycles of 98 °C for 10 s, a primer pair specific annealing temperature (Additional file 3: Table S2) for 30 s and 72 °C for 30 s, and a final elongation at 72 °C for 5 min. PCR products were analysed in 1.5% agarose gels stained with GR Green (Labgene, Chatel-Saint-Denis, Switzerland).

PCR products were purified using the DNA Clean & Concentrator™ kit (Zymo Research, Freiburg, Germany) and submitted to LGC Genomics (Berlin, Germany) for sequencing. Results were analysed using BLASTn [53].

### Pyrosequencing assays

Allele frequencies at codons 167 (TTC/TAC), 198 (GAA/GCA) and 200 (TTC/TAC) were measured using pyrosequencing assays. The assays followed a previously described protocol [26] as modified by Ademola et al. [33]. DNA from pooled adult male *H. contortus* (*n* = 8) collected after albendazole treatment was used to amplify a partial isotype 1 β-tubulin fragment using a biotinylated reverse primer (Additional file 3: Table S2). The total volume of each reaction was 50 μl in 1× buffer HF containing 0.2 mM dNTPs, 0.25 µM of each primer (Additional file 3: Table S2), 1 U Phusion II High Fidelity DNA polymerase (Thermo Fisher Scientific) and 1 μl of template DNA (L3: 10–20 ng; and adult: ~ 125 ng). The cycling conditions were 98 °C initial denaturation for 30 s followed by 40 cycles of 98 °C for 10 s, 56 °C for 30 s and 72 °C for 30 s. The reaction was terminated after incubation at 72 °C for 5 min. Allele frequencies were measured using the PyroMark Q24 system and PyroMark Gold Q24 reagents (Qiagen, Hilden, Germany) and the sequencing primers Hc167PySeq1 (5′-ATA GAA TTA TGG CTT CGT-3′), Haemcon198Seq-Pr (5′-GGT AGA GAA CAC CGA TG-3′) and Hc200PySeq1 (5′-TAG AGA ACA CCG ATG AAA CAT-3′).

### Sanger sequencing of *H. contortus* isotype 1 β-tubulin gene fragments

The same PCR was used for amplification of a partial *H. contortus* isotype 1 β-tubulin gene fragment as for pyrosequencing but with a non-biotinylated reverse primer. PCR products were purified and sent for Sanger sequencing. Sequencing results were submitted to BLASTn searches and analysed in BioEdit software version 7.2.6 [54] to visualise sequence chromatograms to manually inspect them for the presence of SNPs at codons 167, 198 and 200.

## Results

### Albendazole efficacy based on faecal egg count and egg hatch test data

Results of the FECRT and EC_50_ values in the EHT with 95% CIs are presented in Table 1, while Additional file 4: Table S3 shows mean egg count data with 95% CIs. The FECRT results for the 10 mg/kg dose in Rehed Al-Birdi was found to be inconclusive on day 8 and indicate lack of efficacy on day 14; no matter if the results of the treated group were compared with a control group on the same day or the epg of the same animals before treatment. However, the FECRT result for the 10 mg/kg dose treatment was inconclusive in Tulus on day 8 and 14 with unpaired statistics and the treatment was considered not effective when the paired data from treated goats analysed before and after treatment were compared. The FECRT data for the 5 mg/kg dose treatment was inconclusive on day 8 and the treatment was considered not effective based on data from day 14 in both study areas with unpaired and paired results. The retreatment of goats in Tulus on day 14 with a second 5 mg/kg dose albendazole was considered ineffective on days 8 and 14 post-retreatment. The 7.5 mg/kg dose in Rehed Al-Birdi was considered not effective only on day 14 with unpaired results. The highest tested dose, 12.5 mg/kg, was found to be effective, but still 100% efficacy was not achieved (97.5%). In both study areas, EC_50_ values for thiabendazole in the EHT were slightly lower than 0.1 µg/ml thiabendazole (0.08 µg/ml thiabendazole in both study areas) but the upper limits of the 95% CIs were in most cases higher than 0.1 µg/ml and results of the EHT were therefore considered to be inconclusive.

**Table 1:** Results of the faecal egg count reduction (FECR) after treatment with different doses of albendazole and the 50% effective concentration (EC_50_) in the egg hatch test for goats naturally infected with gastrointestinal nematodes or experimentally infected with local *Haemonchus contortus* isolates at Rehed Al-Birdi, Tulus and Um Dafuq (South Darfur, Sudan)

Study area	Infection type	GI nematodes	Dose (mg/kg bw)	No. of animals in each trial	Day 8		Day 14		EC_50_ (µg/ml thiabendazole)
					FECR (%) Unpaired^a^	FECR (%) Paired^a^	FECR (%) Unpaired^a^	FECR (%) Paired^a^	
Rehed Al-Birdi	Natural	Strongyles	5	C: *n* = 8	93.3 (82.1–97.7)	94.1 (92.4–95.6)	87.5 (68.5–94.9)	90.2 (88.3–91.8)	0.08 (0.03–0.24)
				T: *n* = 8					
		Strongyles	7.5	C: *n* = 13	92.1 (80.5–96.6)	95.6 (94.8–96.3)	87.1 (72.8–94.4)	93.7 (92.9–94.5)	
				T: *n* = 10					
		Strongyles	10	C: *n* = 13	90.4 (82–95.2)	92.2 (91.5–93)	86.5 (71.8–93.1)	89.8 (88.9–90.7)	
				T: *n* = 25					
		Strongyles	12.5	C: *n* = 13	97.1 (93.4–98.8)	97.5 (96.7–98)	96.6 (89.9–98.8)	97.2 (96.4–97.8)	
				T: *n* = 13					
Tulus	Natural	Strongyles	5	C: *n* = 11	92 (86.1–95.2)	94.6 (94.1–95)	85.8 (72.1–93.4)	88.5 (87.8–89.1)	0.08 (0.07–0.1)
				T: *n* = 27					
			5	R^b^: *n* = 12	na	64.5 (60.2–68.4)	na	54.4 (49.8–59.4)	
			10	C: *n* = 11	89.9 (76.1–95.2)	90 (88.7–91.2)	89.6 (65.5–96.2)	87.3 (85.8–88.7)	
				T: *n* = 12					
	Experi-mental	*H. contortus*	5	C: *n* = 8	85.2 (60.1–93.5)	86.2 (84.7–87.5)	76.3 (34.1–91.9)	81.8 (79.9–83.4)	0.11 (0.08–0.16)
				T: *n* = 8					
Um Dafuq	Experi-mental	*H. contortus*	5	C: *n* = 8	90.9 (80.2–94.8)	84.2 (82.9–85.5)	82.8 (57–90.8)	73.5 (72–75.4)	0.11 (0.07–0.17)
				T: *n* = 8					

^a^FECRs were calculated either by comparing data post-treatment between treatment and control group (unpaired) or between data post- and pre-treatment (paired); 95% confidence interval is provided in parentheses

^b^Retreated goats were initially treated first with 5 mg/kg albendazole and received a repeated dose of albendazole (5 mg/kg body weight) on day 14

*Abbreviations*: bw, body weight; C, control; T, treated; R, retreated; na, no control group available since the treated group was retreated for the second time

The FECRT results with 95% CI for the experimental infection with *H. contortus* isolates from Tulus and Um Dafuq together with the results of the EHT with 95% CI are also presented in Table 1 and Additional file 4: Table S3. The 5 mg/kg dose of albendazole was not effective in the treatment of male goats infected with both *H. contortus* isolates, no matter if post-treatment samples were collected on days 8 or 14 or the data was analysed with unpaired or paired methods. The EC_50_ value of 0.11 µg/ml thiabendazole in both isolates was indicative of BZ resistance and as such supported the above findings.

### Genus-specific PCRs

Thirty-eight L3 samples collected pre- and post-treatment had good DNA quality (13 samples from day 0 and 25 after treatment). The samples, from Kass, Nyala (Domaia and Majok), Rehed Al-Birdi and Tulus, South Darfur, were analysed with genus-specific PCRs to detect *Haemonchus* spp., *Trichostrongylus* spp., *Cooperia* spp., *Teladorsagia* spp. and *Ostertagia* spp. (Table 2). While *Teladorsagia* spp. and *Ostertagia* spp. were not detected in any of the samples, all pre-treatment samples were positive for *Haemonchus* spp. and *Trichostrongylus* spp. *Cooperia* spp. was detected in 7 out of 13 samples with all negative samples coming from Rehed Al-Birdi in autumn 2016. Post-treatment samples also contained all three genera but with different frequencies than before treatment. *Haemonchus* spp. was still present in all samples. *Trichostrongylus* spp. was completely eliminated in post-treatment pools from Tulus whereas in other regions several pools were still positive for *Trichostrongylus* spp., particularly but not exclusively if the 5 mg/kg dosage was applied. The results for *Cooperia* spp. were very similar with incomplete elimination and better results with the higher dosage.

**Table 2:** Detection of trichostrongyloid genera in third-stage larvae obtained from faecal samples pooled on farm level in different areas in South Darfur before and after treatment with albendazole, using genus-specific PCR

Study area	Season	Dose (mg/kg bw)	*n*^a^	No. of positive pools		
				*Haemonchus*	*Trichostrongylus*	*Cooperia*
Kass	Autumn	0	1	1	1	1
		5	6	6	3	3
		10	1	1	0	0
Nyala Domaia	Autumn	0	1	1	1	1
		5	1	1	1	0
		10	1	1	1	0
	Winter	0	1	1	1	1
		10	2	2	0	1
Nyala Majok	Winter	0	1	1	1	1
		10	1	1	1	1
Rehed Al-Birdi	Autumn	0	7	7	7	1
		5	1	1	0	0
		7.5	1	1	1	1
		10	4	4	1	0
		12.5	1	1	0	0
Tulus	Autumn	0	2	2	2	2
		5	3	3	0	2
		5 + 5^b^	2	2	0	0
		10	1	1	0	0
Total no. of pre-treatment samples (%)			13	13 (100)	13 (100)	7 (54)
Total no. of post-treatment samples (%)			25	25 (100)	8 (32)	8 (32)
Total no. of pre- and post-treatment samples (%)			38	38 (100)	21 (55)	15 (40)

^a^Number of pools tested

^b^Goats treated with 5 mg/kg dose were retreated with the same dose on day 14

*Abbreviation*: bw, body weight

### Genotypes of isotype 1 β-tubulin gene of *H. contortus*

**Fig. 1:**
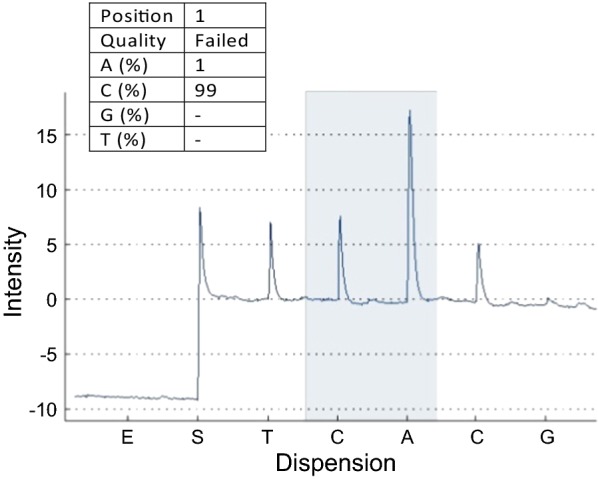
Representative pyrograms for phenotypically benzimidazole resistant *Haemonchus contortus* isotype 1 β-tubulin gene SNPs at position of codon position 198. Pyrograms show signal intensity on the ordinate and the dispersion pattern on the ordinate. *Abbreviations*: E, enzyme; S, substrate; A, dATP; C, dCTP; G, dGTP; T, dTTP

The frequencies of resistance-associated genotypes at codons 167, 198 and 200 were measured in pools of adult male *H. contortus* that survived treatment with albendazole. Pyrosequencing assays detected BZ resistance allele-associated frequencies (TAC) in codon 167 of ≤ 3.6 ± 1.1% and in codon 200 of ≤ 7.4 ± 3.1% (Table 3). In contrast, pyrosequencing failed for codon 198 due to the fact that the surrounding reference peak pattern did not match to the expected sequence and the signal intensity was very low although exactly the same PCR products worked well in codon 167 and codon 200 assays (Fig. 1).

**Table 3:** Mean frequencies (%) ± standard deviation (SD) of benzimidazole resistance associated alleles in isotype 1 β-tubulin gene of *Haemonchus contortus* isolates from South Darfur, Sudan, measured by pyrosequencing

Isolate	Codon 167 (TAC)	Codon 198 (GCA)	Codon 200 (TAC)
Kass	3.3 ± 0.5	Failed	6.8 ± 1.0
Nyala	4.5 ± 1.3	Failed	10.8 ± 4.0
Tulus	3.3 ± 0.5	Failed	6.0 ± 2.0
Um Dafuq	3.3 ± 1.5	Failed	6.5 ± 2.4
Total	3.6 ± 1.1	Failed	7.4 ± 3.1

*Notes*: The samples were pooled adult male *H. contortus* (11–84 worms/each sample) from goats after treatment with 5 or 10 mg/kg body weight albendazole. For each area, two biological and two technical replicates were analysed

**Fig. 2:**
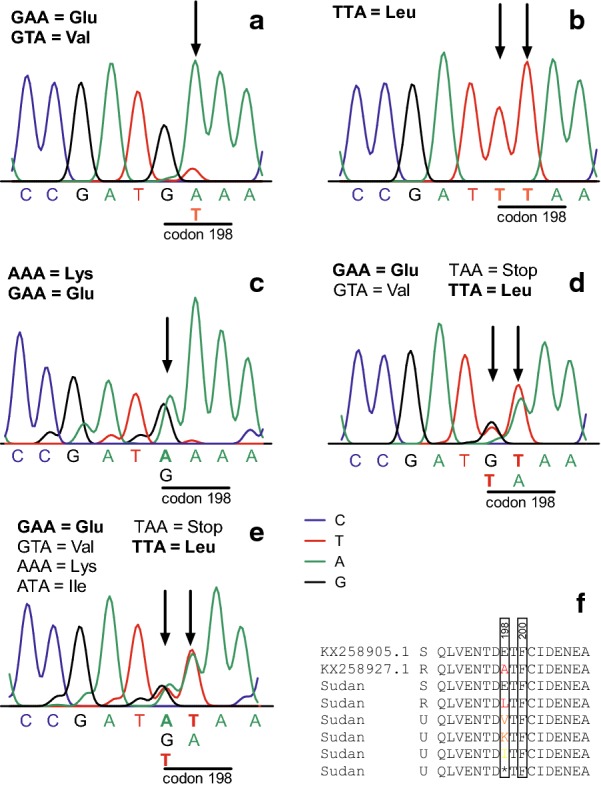
Amino acid changes in codon 198 identified by Sanger sequencing. **a**–**e** Representative chromatograms. Vertical arrows mark polymorphisms in codon position one and/or two. Encoded amino acids are shown above the chromatograms with bold fonts indicated codons/amino acids proven to be present in *Haemonchus contortus* from South Darfur and normal fonts codons/amino acids potentially present. **f** Alignment of amino acids 191–206 with detected and potentially present amino acids in position 198 of isotype 1 β-tubulin gene of *H. contortus* using a sequence from a susceptible (S) isolate (GenBank: KX258905.1), a resistant isolate (GenBank: KX258927.1) and the sequences from the states South, Central and East Darfur, Sudan, with the different codon 198 polymorphisms. U, sequences obtained from samples collected from goats before albendazole treatment or from abattoirs with unknown resistance status. In the alignment, green indicates susceptible and red resistant variants at codon position 198. Variants known to confer benzimidazole resistance in fungi are shown in orange and variants with similar chemical properties as known resistant variants are coloured in yellow

**Fig. 3:**
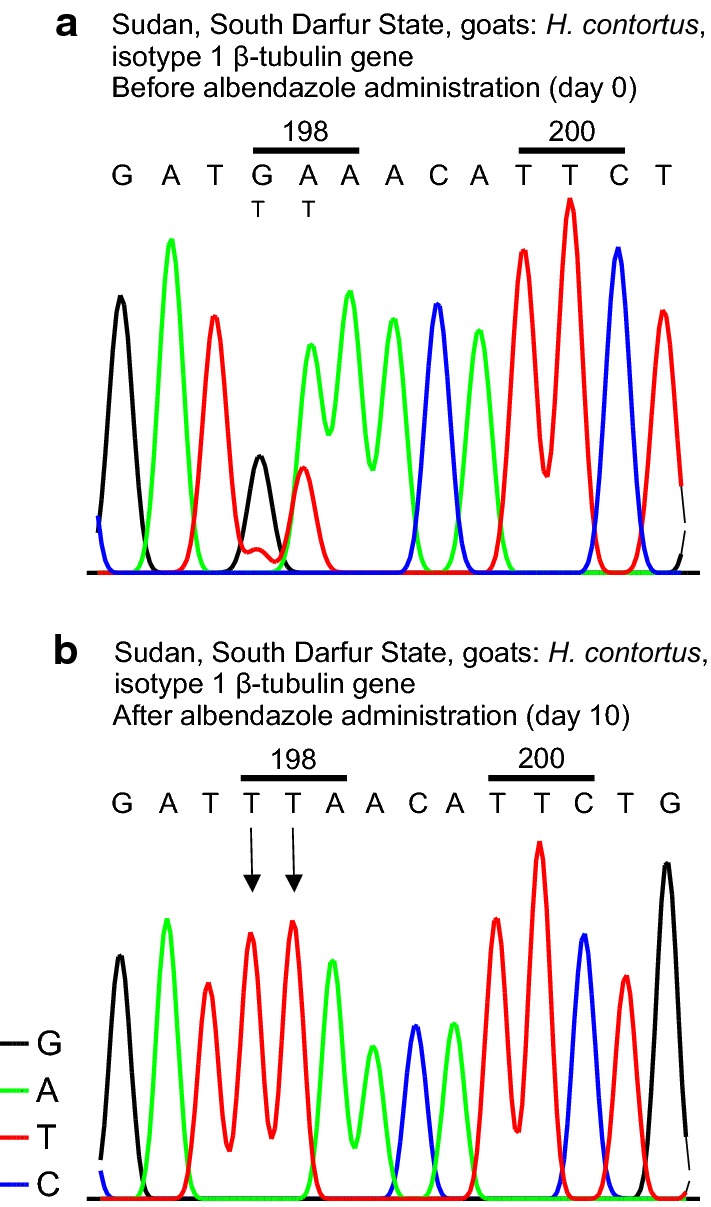
Effects of albendazole treatment on the distribution of TTA (leucine) and GAA (glutamate) at codon position 198. Sequence chromatograms of isotype 1 β-tubulin gene of *Haemonchus contortus* in the regions of codon 198 and 200 using third-stage larvae (L3) samples before and after albendazole administration (paired) in trials of goats in South Darfur. The upper trace (**a**) represents L3 samples before treatment while the lower trace (**b**) corresponds to the L3 obtained after treatment

Since pyrosequencing failed for codon 198 and phenotypically resistant worms contained no high SNP frequencies in codons 167 and 200, conventional Sanger sequencing of PCR products was performed to identify changes that might be involved in resistance. A total of 133 *Haemonchus* spp. samples, pooled L3 (*n* = 36) and pooled adult males (*n* = 97) from all three Darfur States, were successfully genotyped regarding polymorphisms in codons 167, 198 and 200. To increase chances to detect SNPs associated with BZ resistance, 35 samples (L3: *n* = 21 and adults: *n* = 14) collected from goats treated with albendazole at different doses were included, while the remaining samples (*n* = 98) were collected during trials on day 0 (L3: *n* = 15) or from abattoirs (adult: *n* = 83). The latter samples had an unknown history of anthelmintic treatments. All sequences were 99% identical (including intron sequences) to the *H. contortus* isotype 1 β-tubulin gene (GenBank: KX258905). No polymorphisms were found in codons 167 and 200 where all sequences displayed the respective susceptible genotype (TTC/Phe). However, polymorphisms were identified in codon 198 of the isotype 1 β-tubulin gene. These data are presented in Table 4 summarising the presence of SNPs together with sample numbers and origins. For codon 198, the wild type amino acid, glutamate (GAA), was identified in 10.5% of the samples (L3 and adults together, irrespective of the treatment history) (Fig. 2a), while the amino acid alanine (GCA) associated with resistance in previous studies was not identified in any of the samples including those that survived the highest albendazole doses (12.5 mg/kg bw). However, other polymorphisms at codon position 198 were found, namely TTA (leucine) (frequency 82%; Fig. 2b). In some samples, low frequency of GTA (valine) (4.5%; Fig. 2a) and AAA (lysine) (0.8%; Fig. 2c) were clearly identifiable. In a few samples, the patterns could not be unequivocally resolved with more than one base present in both codon position one and two. For instance, the pattern in Fig. 2d might represent a mixture of TTA (leucine), GTA (valine) and GAA (glutamate) but also a TAA stop codon cannot be excluded. Even more complicated is the pattern shown in Fig. 2e, which can be explained by the presence of TTA (leucine) and GAA (glutamate) plus AAA (lysine) and/or ATA (isoleucine). Presence of GTA (valine) or TAA (stop codon) also cannot be excluded. Leucine was detected in 100% of both L3 and adult samples that were collected post-albendazole treatment, no matter if goats had received 5 mg/kg or higher dosages up to 12.5 mg/kg bw. Remarkably, leucine was also detected in 100% of the samples collected at abattoirs from Central and East Darfur without known history of recent anthelmintic treatment. When comparing the sequence chromatograms of paired L3 samples before and after treatment, the intensity of the TTA (leucine) signal strongly increased in post-treatment samples as exemplified in Fig. 3. Valine was encoded in both types of samples, L3 and adults from South Darfur, while lysine and potentially isoleucine and the stop codon TAA were only found in pooled adult samples from abattoirs in South Darfur.

**Table 4:** Polymorphisms in codon 198 in isotype 1 β-tubulin gene of *Haemonchus contortus* collected in different areas in the States South, Central and East Darfur in Sudan detected by Sanger sequencing

State	Area	Sample source	Sample type^a^	Treatment^b^	*n*^c^	No. of samples with each variant						
						GAA	GCA	TTA	GTA	AAA	ATA	TAA
South Darfur	Buram	Abattoir	Adult	na	11	1	0	9	0	0	0	1
	Kass	Trials	L3	−	1	0	0	1	0	0	0	0
				+	7	0	0	7	0	0	0	0
			Adult	+	3	0	0	3	0	0	0	0
		Abattoir	Adult	na	9	0	0	8	1	0	0	0
	Nyala	Trials	L3	−	4	1	0	3	0	0	0	0
				+	4	0	0	4	0	0	0	0
			Adult	+	3	0	0	3	0	0	0	0
		Abattoir	Adult	na	20	2	0	13	4	1	0	0
	Rehed Al-Birdi	Trials	L3	−	8	3	0	5	0	0	0	0
				+	7	0	0	7	0	0	0	0
			Adult	+	2	0	0	2	0	0	0	0
		Abattoir	Adult	na	8	3	0	4	1	0	0	0
	Tulus	Trials	L3	−	2	1	0	1	0	0	0	0
				+	3	0	0	3	0	0	0	0
			Adult	+	4	0	0	4	0	0	0	0
		Abattoir	Adult	na	13	3	0	8	0	0	1	1
	Um Dafuq	Trials	Adult	+	2	0	0	2	0	0	0	0
Central Darfur	Zalingei	Abattoir	Adult	na	11	0	0	11	0	0	0	0
East Darfur	Ed Daein	Abattoir	Adult	na	11	0	0	11	0	0	0	0
Total (and %)					133	14 (10.5)	0 (0)	109 (82)	6 (4.5)	1 (0.8)	1 (0.8)	2 (1.5)

^a^Pools of third-stage larvae (L3) (at least ~ 1000 nematode L3/sample) or of adults (trials: 11–84, abattoirs: 10–97 adult male *H. contortus*/sample)

^b^Third-stage larval samples (L3): (−) faecal samples collected on day 0; (+) the samples collected 10 days after treatment with albendazole at different doses: 5 or 10 mg/kg body weight (bw) (all study areas), and additionally 7.5 or 12.5 mg/kg bw in trials of Rehed Al-Birdi. Adult male *H. contortus*: samples isolated from goats slaughtered on day 14 after treatment with 5 or 10 mg/kg bw albendazole (+)

^c^Number of pools tested

*Abbreviation*: na, the situation of anthelmintic treatments was unknown, since adult male *H. contortus* isolated from goat abomasa at abattoirs

Attempts to design pyrosequencing assays to quantify the E198L polymorphisms failed. Since these polymorphisms differ at codon position 1 and 2 from the wild type and the same nucleotide (T) can be involved at both positions, it is principally not possible to quantify the frequencies of the codons GAA (glutamate), GCA (alanine) and TTA (leucine) using pyrosequencing since assay analyses always failed in the Pyromark Q24 software.

## Discussion

Phenotypic data in a previous and in this study showed that AR is widespread in GINs of goats in Sudan but also identified some susceptible populations. In such a constellation, rapid and cost-effective identification of resistant populations can help to limit spread of resistance, e.g. by implementation of alternative treatment and quarantine schemes. This requires detailed knowledge of resistance mechanisms on the molecular level and field-evaluated molecular diagnostic tools.

For the BZs, polymorphisms in the isotype 1 β-tubulin gene have been shown to confer resistance in strongyle nematodes [55]. In *H. contortus*, the substitutions F167Y, E198A and F200Y have been identified as resistance-associated [16, 18]. These polymorphisms were also identified in other species such as *C. oncophora*, *O. ostertagi* and *T. circumcincta* [7, 19]. The recently described codon 198 mutation (E198L) in *T. circumcincta* was also shown to be widespread at high frequency on many UK farms in *Trichostrongylus axei* [19, 20]. Multiple molecular tools to qualitatively identify or quantify these variants have been developed [20, 24–28].

The present study aimed to understand the BZ-resistance mechanisms in *H. contortus* isolates from Sudan using pyrosequencing assays. While no SNP frequencies higher than the technical background (10% [33]) were detected in codons 167 and 200, pyrosequencing for codon 198 failed. Sanger sequencing identified five novel polymorphisms in codon 198 of the *H. contortus* isotype 1 β-tubulin gene, i.e. E198L, E198V and E198K, as well as potentially E198I and E198Stop. All these substitutions are new for *H. contortus*, and E198I, E198K and E198Stop have never been reported in BZ resistant strongyle nematodes. In contrast, E198L has been reported three times and E198V once in *T. circumcincta* [19, 20, 56, 57]. Whether the only potentially present variants E198I and E198Stop represent true haplotypes or are in fact absent will be investigated using deep amplicon sequencing analyses in the future.

The E198L substitution was present in 82% of all tested samples (*n* = 133) and in 100% of the L3 and pooled adult samples collected post-albendazole treatment. The fact that the frequency of E198L increased in paired samples before and after albendazole treatment is a strong indicator that albendazole treatment selects for this genotype and that leucine at this position of the isotype 1 β-tubulin gene might be sufficient to confer BZ resistance in *H. contortus*. The E198L was shown to be widespread in *T. circumcincta* and *Tr. axei* on UK sheep farms [20] and also identified on a single sheep farm from Ireland with resistant *T. circumcincta* with a frequency of 17% [57] and in a multi-drug resistant *T. circumcincta* isolate [56]. On most farms in the UK and Ireland, the F200Y polymorphism was also present with a much higher frequency. On one UK farm, however, the E198L mutation occurred with a very high frequency of 91.7% [19]. When deep amplicon sequencing was adopted using the same samples where the E198L polymorphism was detected in *T. circumcincta* in UK [19], the frequencies in *T. circumcincta* were 6.4% and 7.8% in pooled faecal samples from ewes and lambs, respectively, while in *H. contortus*, the E198L frequency was 0% [20]. The latter study also detected E198V at very low frequencies. The present study is the first to show that the E198L substitution is relevant in a wider geographical area and can be the most frequent polymorphism in resistant *H. contortus* populations.

**Fig. 4:**
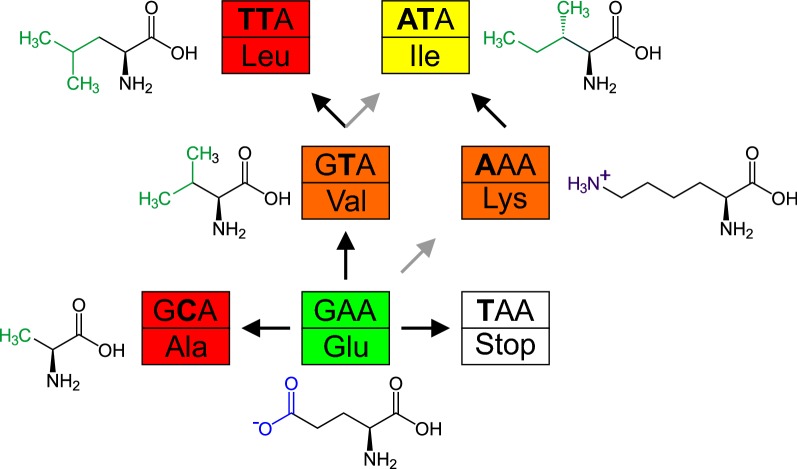
Mutation pathways connecting codon 198 variants found in isotype 1 β-tubulin gene of *Haemonchus contortus*. The susceptible genotype is shown in green, variants known to confer benzimidazole resistance in parasitic nematodes in red, variants implicated in benzimidazole resistance in fungi in orange and variants suspected to confer resistance in due to similarity with other amino acids in yellow. Black and grey arrows indicate transversions and transitions, respectively. In the amino acid structures, blue, dark green and dark violet highlight negatively charged, hydrophobic aliphatic and positively charged groups

In comparison to the well-known E198A substitution caused by one SNP (G**A**A → G**C**A) [18], the situation is more complex for E198L which requires two nucleotide substitutions (**GA**A → **TT**A) in codon positions 1 and 2. Similarly, in a few of the samples isoleucine was potentially encoded, which is also based on two nucleotide substitutions (**GA**A → **AT**A). Interestingly, the second most frequently found non-wild type variant E198V (G**A**A → G**T**A, 4.5%) with a single substitution in the second codon position can be considered to be an intermediate leading to E198I or E198L by a second substitution as discussed by Avramenko et al. [20] for E198L (Fig. 4). Other interesting findings were discovered that are based on a single substitution in the first codon position resulting in lysine (E198K, **G**AA → **A**AA; 0.8%) or in a stop codon (**G**AA → **T**AA, 1.5%) (Fig. 4). However, leucine, valine and isoleucine are all amino acids with hydrophobic, aliphatic side chains and thus are very similar to alanine, which is well known to cause BZ resistance when present in position 198. Therefore, it is very likely that these amino acids have similar effects on BZ binding affinity as alanine. Indeed, E198V has been described to confer BZ resistance in fungi such as *Botrytis cinerea* [22] and E198L was identified in the BZ resistant fungi of the species *Gibberella zeae* [21] and *Fusarium asiaticum* [58]. In contrast, lysine is a positively charged amino acid and thus has very different chemical properties. Therefore, effects of this substitution are difficult to predict. However, multiple polymorphisms have been described in codon 198 of fungal β-tubulin genes from *Aspergillus nidulans* and *Neurospora crassa* resistant to the BZ fungicides such as carbendazim. These polymorphisms included substitutions from glutamate to aspartate (E198D), glutamine (E198Q), glycine (E198G) and the lysine (E198K) that was also found in the present study [21, 58–60]. This suggests that even this polymorphism might be associated with BZ resistance in the field populations from Sudan, but further functional and structural analyses will be required to corroborate this for nematode isotype 1 β-tubulins.

Surprisingly, one of the potentially present variants in codon 198 encoded a stop codon. This missense mutation leads to a truncated and most likely non-functional protein. However, nematode genomes typically encode multiple β-tubulin paralogs with very high sequence identity. Thus, the function of the missing gene might be sufficiently replaced by its paralogs to circumvent lethal effects. According to the sequences in codons 167, 198 and 200, virtually all wild-type β-tubulin paralogs in strongyle nematodes are predicted to be BZ susceptible. Therefore, simple deletion of one paralog would not be predicted to cause BZ resistance. Despite that, a loss-of-function deletion in the *C. elegans ben-1* β-tubulin paralog is well known to cause BZ resistance [61] but this effect occurs in the presence of F200Y in the other major isotypes in *C. elegans*. Moreover, Kwa et al. [62] have shown that high level BZ resistance in *H. contortus* can be associated with deletion of the isotype 2 β-tubulin gene. Thus, although a loss-of-function allele can also contribute to the resistance phenotype in the context of certain genotypes of other β-tubulin paralogs, this is quite unlikely for *H. contortus* as long as there is a functional, BZ susceptible isotype 2 β-tubulin paralog encoded in the genome.

The fact that the E198L genotype cannot be quantified if both wild-type glutamate and the classical resistance-associated variant alanine are also present, or at least should be considered to be present, compromises the codon 198 pyrosequencing assays in general. This also concerns the assay for *T. circumcincta*, a species for which the E198L polymorphism has been described before, albeit most of the time at low frequencies. The fact that E198L was the predominant genotype in the present study caused complete failure of data analysis in pyrosequencing and focused the attention of the authors on the problem. If E198L is only present in low frequencies and E198A is also present, the discrepancies of signal patterns might still be within the tolerance range of the Pyromark software before it reports problems. If this is the case, wrong frequencies, i.e. higher level of the susceptible allele, would be reported and resistant populations might be overlooked. In this context, it would also be advisable to compare Pyromark software versions for Q96, Q48 and Q24 sequencers if they respond in the same way if E198L is present in addition to other variants.

The FECRT data in the present study were analysed on day 8 and day 14 and paired analyses was compared to unpaired comparison of a treatment with a control group. The current WAAVP guideline on detection of anthelmintic resistance [43] recommends analysing samples 8–10 days after BZ treatment or 14 days if other drug classes are analysed in parallel. It also describes both paired and unpaired study designs. The upcoming new (unpublished) WAAVP guideline as presented at the WAAVP conference 2019 [63] will promote later sampling such as 10–14 days post-treatment also for the BZs and also paired study design. Considering these suggested changes in recommendations, it is important to see that indeed paired study design and late sampling increased the number of cases in which resistance was detected while the number of comparisons with ambiguous results decreased. This difference was not evaluated statistically since the number comparison to expect meaningful results is too small but the tendency is important to note and it would be of interest to further evaluate the effects of study design and day of resampling on the sensitivity to detect anthelmintic resistance in a meta-analysis pooling data from several studies.

In the present study, genus-specific PCRs were also used to allow highly sensitive qualitative diagnosis of *Haemonchus* spp., *Trichostrongylus* spp., *Cooperia* spp., *Teladorsagia* spp. and *Ostertagia* spp. in L3 samples collected before and after treatment in South Darfur. This approach helped to improve understanding of albendazole efficacy when the 5 mg/kg dose, as recommended for goats in Sudan, was compared to 7.5, 10 and 12.5 mg/kg bw doses. In day 0 samples, *Haemonchus* spp., *Trichostrongylus* spp. and *Cooperia* spp. but no *Teladorsagia* spp. and *Ostertagia* spp. were detected. In previous studies regarding GINs from Sudan using *post-mortem* worm recovery, the two latter genera were also not reported in sheep and goats [64, 65]. As expected and in agreement with previous results obtained by morphological differentiation of L3 [66, 67], *H. contortus* was detected in 100% of L3 samples collected after albendazole treatment in the four study areas, no matter if goats were treated with 5 mg/kg or if the dose was increased up to 12.5 mg/kg bw. In contrast to previous reports using larval cultures and morphological differentiation, in the present study, based on molecular identification *Trichostrongylus* spp. and *Cooperia* spp. were also detected in L3 samples from treated goats in the four study areas (Table 2) which can be explained by the higher sensitivity of the PCR. However, the quantitative data obtained by morphological differentiation of larvae indicate that the vast majority of egg-shedding after treatment was due to *H. contortus*. Changing the albendazole dose from 5 to 10 mg/kg bw was successful in removing *Trichostrongylus* spp. and *Cooperia* spp. in Tulus and in removing *Cooperia* spp. in Rehed Al-Birdi. However, the presence of *Trichostrongylus* spp. and *Cooperia* spp. in L3 samples from goats treated with 10 mg/kg in Kass and Nyala suggests that evolution of BZ resistance in these nematodes has also occurred. This suggestion is supported by reports from three different African regions (Ethiopia, Mozambique and Zimbabwe) which detected *Trichostrongylus* spp. and *Cooperia* spp. in faecal samples from goats treated with albendazole [42, 68, 69].

## Conclusions

Benzimidazole resistance in *H. contortus* was widely distributed in South Darfur, Sudan, but BZ-resistant *Cooperia* spp. and *Trichostrongylus* spp. were also detected. In *H. contortus*, resistance was associated with a widely distributed E198L substitution in isotype 1 β-tubulin gene that was present in all post-treatment samples. Additional new polymorphisms at codon position 198 encoding valine, isoleucine and lysine are also likely to confer resistance while a TAA stop codon at this position is predicted to cause resistance only if additional changes in the isotype 2 gene are present. Functional and structural studies regarding the effects of the five new substitutions in model systems will be required to understand the exact molecular interactions leading to BZ resistance.

## Supplementary information

**Additional file 1: Figure S1.** Map of Sudan showing the location of the three States of Darfur (Central, East and South Darfur) and the areas where field trials and abattoir samples collected: 1, Buram (10.85°N, 25.00°E); 2, Ed Daein (11.26°N, 26.09°E); 3, Kass (12.50°N, 24.28°E); 4, Nyala (12.05°N, 24.88°E); 5, Rehed Al-Birdi (11.30°N, 23.88°E); 6, Tulus (11.00°N, 2.00°E); 7, Um Dafuq (10.41°N, 23.41°E); and 8, Zalingei (12.54N, 23.28°E). The map of the eight study areas was generated using Tableau desktop professional software for windows version 2018.1.1. Minor modifications were made using Paint software for windows version 1803.

**Additional file 2: Table S1.** Overview of study design and sample origin.

**Additional file 3: Table S2.** Primers used for PCR.

**Additional file 4: Table S3.** Arithmetic means (95% confidence interval) of egg counts for goats naturally infected with gastrointestinal nematodes or experimentally infected with *Haemonchus contortus* in three different South Darfur (Sudan) study areas before and after oral administration of albendazole at different doses to the treated groups.

## Data Availability

All relevant information has been included in the manuscript and its additional files. Data analysed in this study are available from the corresponding author upon request. The partial sequence of isotype 1 β-tubulin gene of *H. contortus* Sudan isolates, with codon 198 substitution (E198L), was submitted to the GenBank database under the Accession number MN657178.

## Abbreviations

AR: anthelmintic resistance
BZs: benzimidazoles
bw: body weight
CIs: confidence intervals
EC_50_: concentration of thiabendazole that inhibited 50% of larvae hatching
EHT: egg hatch test
epg: eggs per gram
FECRT: faecal egg count reduction test
GINs: gastrointestinal nematodes
L3: third-stage larvae
SNPs: single nucleotide polymorphisms
WAAVP: World Association for the Advancement of Veterinary Parasitology
