# Supplementary material for: New codon 198 β-tubulin polymorphisms in highly benzimidazole resistant Haemonchus contortus from goats in three different states in Sudan
Source: Parasit Vectors. 2020 Mar 2;13:114. doi: 10.1186/s13071-020-3978-6 (PMC7053126; doi:10.1186/s13071-020-3978-6)
Supplement: Supplementary file 1 — Additional file 1: Figure S1. Map of Sudan showing the location of the three States of Darfur (Central, East and South Darfur) and the areas where field trials and abattoir samples collected: 1, Buram (10.85°N, 25.00°E); 2, Ed Daein (11.26°N, 26.09°E); 3, Kass (12.50°N, 24.28°E); 4, Nyala (12.05°N, 24.88°E); 5, Rehed Al-Birdi (11.30°N, 23.88°E); 6, Tulus (11.00°N, 2.00°E); 7, Um Dafuq (10.41°N, 23.41°E); and 8, Zalingei (12.54N, 23.28°E). The map of the eight study areas was generated using Tableau desktop professional software for windows version 2018.1.1. Minor modifications were made using Paint software for windows version 1803. [file 13071_2020_3978_MOESM1_ESM.pdf]

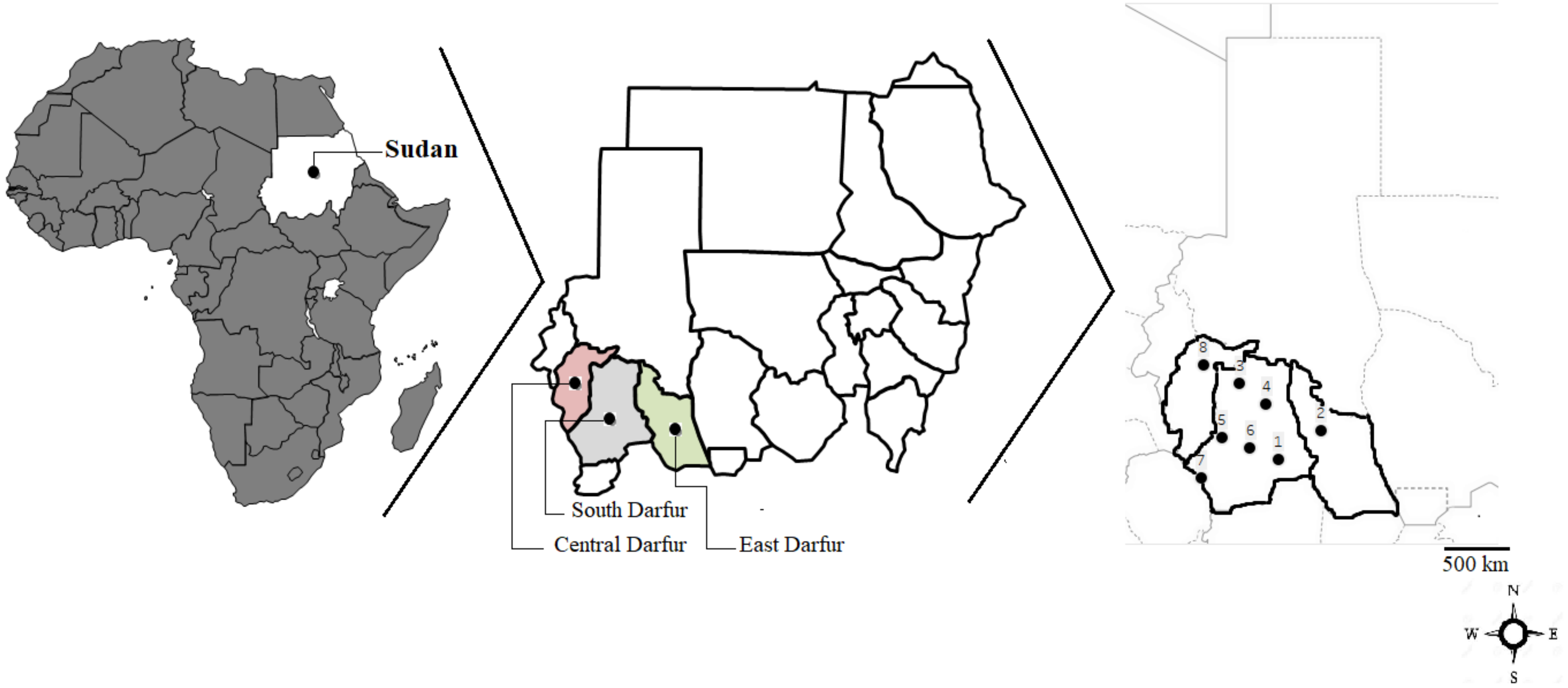

Fig. S1. Map of Sudan showing the location of the three States of Darfur (Central, East and South Darfur) and the areas where field trials and abattoir samples collected. The areas were 1. Buram (10.85°N 25.00°E), 2. Ed Daein (11.26°N 26.09°E), 3. Kass (12.50°N 24.28°E), 4. Nyala (12.05°N 24.88°E), 5. Rehed Al-Birdi (11.30°N 23.88°E), 6. Tulus (11.00°N 2.00°E), 7. Um Dafuq (10.41°N 23.41°E) and 8. Zalingei (12.54N 23.28°E). The map of the eight study areas was generated using Tableau desktop professional software for windows version 2018.1.1. Minor modifications were made using Paint software for windows version 1803.
