# Supplementary material for: New codon 198 β-tubulin polymorphisms in highly benzimidazole resistant Haemonchus contortus from goats in three different states in Sudan
Source: Parasit Vectors. 2020 Mar 2;13:114. doi: 10.1186/s13071-020-3978-6 (PMC7053126; doi:10.1186/s13071-020-3978-6)
Supplement: Supplementary file 2 — Additional file 2: Table S1. Overview of study design and sample origin. [file 13071_2020_3978_MOESM2_ESM.docx]

**Additional file 2: Table S1** Overview of study design and sample origin

| **State** | **Region** | **Infection type** | **Sample source** | **Experiment** | **Material** | **Pooling level** | **Tested dose of albendazole (mg/kg bw)** | **Reference** |
| --- | --- | --- | --- | --- | --- | --- | --- | --- |
| South Darfur | Buram | Natural | Abattoir | Molecular analyses | Adults | Animal^c^ | na | Present study |
|  | Kass | Natural | Trial | FECRT | Eggs | Region | 5 and 10 | [34] |
|  |  |  | Trial | EHT | Eggs | Region | na | [34] |
|  |  |  | Trial | Molecular analyses | L3 | Farm | 5 and 10 | Present study |
|  |  |  | Trial | Molecular analyses | Adults | Animal^c^ | 10 | Present study |
|  |  | Experimental^a^ | Trial | FECRT | Eggs | Region^b^ | 5 and 10 | [34] |
|  |  |  | Trial | EHT | Eggs | Region^b^ | na | [34] |
|  |  |  | Trial | Molecular analyses | Adults | Animal^c^ | 10 | Present study |
|  |  | Natural | Abattoir | Molecular analyses | Adults | Animal^c^ | na | Present study |
|  | Nyala | Natural | Trial | FECRT | Eggs | Region | 5, 5 twice and 10 | [34] |
|  |  |  | Trial | EHT | Eggs | Region | na | [34] |
|  |  |  | Trial | Molecular analyses | L3 | Farm | 5, 5 twice and 10 | Present study |
|  |  |  | Trial | Molecular analyses | Adults | Animal^c^ | 10 | Present study |
|  |  | Experimental^a^ | Trial | FECRT | Eggs | Region^b^ | 5 | [34] |
|  |  |  | Trial | EHA | Eggs | Region^b^ | na | [34] |
|  |  |  | Trial | Molecular analyses | Adults | Animal^c^ | 5 | Present study |
|  |  | Natural | Abattoir | Molecular analyses | Adults | Animal^c^ | na | Present study |
|  | Rehed Al-Birdi | Natural | Trial | FECRT | Eggs | Region | 5, 7.5, 10 and 12.5 | Present study |
|  |  |  | Trial | EHT | Eggs | Region | na | Present study |
|  |  |  | Trial | Molecular analyses | L3 | Farm | 5, 7.5, 10 and 12.5 | Present study |
|  |  |  | Trial | Molecular analyses | Adults | Animal^c^ | 5 and 10 | Present study |
|  |  | Natural | Abattoir | Molecular analyses | Adults | Animal^c^ | na | Present study |
|  | Tulus | Natural | Trial | FECRT | Eggs | Region | 5, 5 twice and 10 | Present study |
|  |  |  | Trial | EHT | Eggs | Region | na | Present study |
|  |  |  | Trial | Molecular analyses | L3 | Farm | 5, 5 twice and 10 | Present study |
|  |  |  | Trial | Molecular analyses | Adults | Animal^c^ | 5 and 10 | Present study |
|  |  | Experimental^a^ | Trial | FECRT | Eggs | Region^b^ | 5 | Present study |
|  |  |  | Trial | EHT | Eggs | Region^b^ | na | Present study |
|  |  |  | Trial | Molecular analyses | Adults | Animal^c^ | 5 | Present study |
|  |  | Natural | Abattoir | Molecular analyses | Adults | Animal^c^ | na | Present study |
|  | Um Dafuq | Experimental^a^ | Trial | FECRT | Eggs | Region^b^ | 5 | Present study |
|  |  |  | Trial | EHT | Eggs | Region^b^ | na | Present study |
|  |  |  | Trial | Molecular analyses | Adults | Animal^c^ | 5 | Present study |
| Central Darfur | Zalingei | Natural | Abattoir | Molecular analyses | Adults | Animal^c^ | na | Present study |
| East Darfur | Ed Daein | Natural | Abattoir | Molecular analyses | Adults | Animal^c^ | na | Present study |

^a^*Haemonchus contortus* were isolated from 50 naturally infected goats from the abattoir of each study area and prepared for experimental infection trials

^b^Since the origin of the infected materials were from the abattoir, the pooling level was cited as a region

^c^All adult male *H. contortus* in each abomasum were isolated and pooled to be used for molecular analyses

5 twice, Goats were initially treated first with 5 mg/kg body weight albendazole and received a repeated dose of albendazole (5 mg/kg) on day 14

*Abbreviations*: bw, body weight; na, the history of last anthelmintic treatment was either more than one month (natural infection trials) or unknown as for the samples isolated from abattoir including materials used for experimental infection trials
