# Supplementary material for: New codon 198 β-tubulin polymorphisms in highly benzimidazole resistant Haemonchus contortus from goats in three different states in Sudan
Source: Parasit Vectors. 2020 Mar 2;13:114. doi: 10.1186/s13071-020-3978-6 (PMC7053126; doi:10.1186/s13071-020-3978-6)
Supplement: Supplementary file 3 — Additional file 3: Table S2. Primers used for PCR. [file 13071_2020_3978_MOESM3_ESM.docx]

**Additional file 3: Table S2** Primers pairs used for PCR

| **Target gene** | **Target parasite** | **Primer sequence (**5´ → 3´) | **(ºC)^a^** | **Length (bp)** |
| --- | --- | --- | --- | --- |
| ITS-2 | *Haemonchus* spp. | F: CCATATACTACAATGTGGCTAATTTC  R: TACAAATGATAAAAGAACATCGTCGC | 62 | 226 |
|  | *Trichostrongylus* spp. | F: CTTACGTCTGGTTCAGGGTTG  R: ACTGAAATGGGAATCATCACAATATTT | 53 | 106 |
|  | *Cooperia* spp. | F: ATGGCATTTGTCTACATCTGTTT  R: AAATGATAACGAATACTACTATCTCCA | 62 | 192 |
|  | *Teladorsagia* spp. | F: AACATATGCAACATGACGTACGACGG  R: ATGATACATTGAACATATATTACCATACATGTCT | 53 | 147 |
|  | *Oestertagia* spp. | F: TAACATTGTTAACGTTACTGAATGATACTG  R: ATATAAATGATACATCGAATATACAATAC | 50 | 124 |
| Isotype 1 β-tubulin | *Haemonchus contortus* | F: GACGCATTCACTTGGAGGAG  R: CATAGGTTGGATTTGTGAGTT | 56 | 386 |

^a^Annealing temperatures

*Abbreviations*: bp, base pair; F, forward primer; R, reverse primer
