# Supplementary material for: New codon 198 β-tubulin polymorphisms in highly benzimidazole resistant Haemonchus contortus from goats in three different states in Sudan
Source: Parasit Vectors. 2020 Mar 2;13:114. doi: 10.1186/s13071-020-3978-6 (PMC7053126; doi:10.1186/s13071-020-3978-6)
Supplement: Supplementary file 4 — Additional file 4: Table S3. Arithmetic means (95% confidence interval) of egg counts for goats naturally infected with gastrointestinal nematodes or experimentally infected with Haemonchus contortus in three different South Darfur (Sudan) study areas before and after oral administration of albendazole at different doses to the treated groups. [file 13071_2020_3978_MOESM4_ESM.docx]

**Additional file 4: Table S3** Arithmetic means (95% confidence interval) of egg counts for goats naturally infected with gastrointestinal nematodes or experimentally infected with *Haemonchus contortus* in three different South Darfur (Sudan) study areas before and after oral administration of albendazole at different doses to the treated groups

| **Study area** | **Infection type** | **GI nematodes** | **Dose (mg/kg bw)** | **Groups** | **Mean (95% CI)** | | |
| --- | --- | --- | --- | --- | --- | --- | --- |
|  |  |  |  |  | **Day 0** | **Day 8** | **Day 14** |
| Rehed Al-Birdi | Natural | Strongyles | 5 | C: *n* = 8 | 1680 (958–2402) | 1620 (747–2493) | 1525 (608–2442) |
|  |  |  |  | T: *n* = 8 | 1735 (1052–2418) | 65^*#^ (6–124) | 163 (29–296) |
|  |  | Strongyles | 7.5 | C: *n* = 13 | 2158 (1080–3237) | 2089 (1272–2906) | 2126 (1235–3017) |
|  |  |  |  | T: *n* = 10 | 3816 (1844–5788) | 136^*#^ (-35–307) | 240^*#^ (86–394) |
|  |  | Strongyles | 10 | C: *n* = 13 | 2158 (1080–3237) | 2089 (1272–2906) | 2126 (1235–3017) |
|  |  |  |  | T: *n* = 25 | 2544 (1680–3408) | 173^*#^ (92–254) | 219^*#^ (89–350) |
|  |  | Strongyles | 12.5 | C: *n* = 13 | 2158 (1080–3237) | 2089 (1272–2906) | 2126 (1235–3017) |
|  |  |  |  | T: *n* = 13 | 3262 (2103–4420) | 43^*#^ (9–78) | 49^*#^ (-9–108) |
| Tulus | Natural | Strongyles | 5 | C: *n* = 11 | 4607 (2226–6988) | 3944 (1201–6687) | 5044 (1979–8108) |
|  |  |  |  | T: *n* = 27 | 4824 (3678–5970) | 213^*#^ (131–295) | 521^*#^ (289–753) |
|  |  | Strongyles | 5 | R^ab^: *n* = 12 | 913 (509–1318) | 310 (101–519) | 413 (153–673) |
|  |  | Strongyles | 10 | C: *n* = 11 | 4607 (2226–6988) | 3944 (1201–6687) | 5044 (1979–8108) |
|  |  |  |  | T: *n* = 12 | 5720 (2659–8781) | 213^*#^ (59–368) | 272^*#^ (-29–573) |
|  | Experimental | *H. controtus* | 5 | C: *n* = 8 | 3765 (1948–5582) | 4480 (1112–7848) | 4218 (228–8207) |
|  |  |  |  | T: *n* = 8 | 3715 (2298–5132) | 495^*#^ (169–821) | 655 (158–1152) |
| Um Dafuq | Experimental | *H. controtus* | 5 | C: *n* = 8 | 4685 (1640–7730) | 13000 (6727–19273) | 11736 (5847–17625) |
|  |  |  |  | T: *n* = 8 | 5383 (2397–8368) | 845^*#^ (553–1137) | 1418^*#^ (866–1969) |

^a^Retreated goats were initially treated first with 5 mg/kg albendazole and received a repeated dose of albendazole (5 mg/kg body weight) on day 14

^b^No control group available, since the treated group was retreated for the second time

*Significantly different (*P* < 0.05) to control on the same day using a Kruskal-Wallis test with Dunn’s post hoc test

^#^Significantly different (*P* < 0.05) to day 0 in the same group using a Friedman test with Dunn’s post hoc test

⸆Significantly different (*P* < 0.05) to day 8 in the same group using a Friedman test with Dunn’s post hoc test

*Abbreviations*: bw, body weight; C, control; T, treated; R, retreated
